# The Effect of Histopathological Growth Patterns of Colorectal Liver Metastases on the Survival Benefit of Adjuvant Hepatic Arterial Infusion Pump Chemotherapy

**DOI:** 10.1245/s10434-023-14342-1

**Published:** 2023-10-02

**Authors:** W. F. Filipe, Y. M. Meyer, F. E. Buisman, R. R. J. Coebergh van den Braak, B. Galjart, D. J. Höppener, W. R. Jarnagin, N. E. Kemeny, T. P. Kingham, P. M. H. Nierop, E. P. van der Stok, D. J. Grünhagen, P. B. Vermeulen, B. Groot Koerkamp, C. Verhoef, M. I. D’Angelica

**Affiliations:** 1 Department of Medical Oncology, Erasmus MC Cancer Institute, Erasmus University Medical Center, Rotterdam, The Netherlands; 2 Department of Surgery, Memorial Sloan Kettering Cancer Center, New York, NY USA; 3 Department of Medical Oncology, Memorial Sloan Kettering Cancer Center, New York, NY USA; 4 Translational Cancer Research Unit (GZA Hospitals and University of Antwerp), Antwerp, Belgium; 5 Department of Surgery, Erasmus MC Cancer institute, Rotterdam, The Netherlands

## Abstract

**Background:**

Histopathological growth patterns (HGPs) are a prognostic biomarker in colorectal liver metastases (CRLM). Desmoplastic HGP (dHGP) is associated with liver-only recurrence and superior overall survival (OS), while non-dHGP is associated with multi-organ recurrence and inferior OS. This study investigated the predictive value of HGPs for adjuvant hepatic arterial infusion pump (HAIP) chemotherapy in CRLM.

**Methods:**

Patients undergoing resection of CRLM and perioperative systemic chemotherapy in two centers were included. Survival outcomes and the predictive value of HAIP versus no HAIP per HGP group were evaluated through Kaplan–Meier and Cox regression methods, respectively.

**Results:**

We included 1233 patients. In the dHGP group (*n *= 291, 24%), HAIP chemotherapy was administered in 75 patients (26%). In the non-dHGP group (*n *= 942, 76%), HAIP chemotherapy was administered in 247 patients (26%). dHGP was associated with improved overall survival (OS, HR 0.49, 95% CI 0.32–0.73, *p *< 0.001). HAIP chemotherapy was associated with improved OS (HR 0.61, 95% CI 0.45–0.82, *p *< 0.001). No interaction could be demonstrated between HGP and HAIP on OS (HR 1.29, 95% CI 0.72–2.32, *p *= 0.40).

**Conclusions:**

There is no evidence that HGPs of CRLM modify the survival benefit of adjuvant HAIP chemotherapy in patients with resected CRLM.

Up to 70% of patients experience recurrence of disease after hepatic resection for colorectal liver metastases (CRLM), despite advances in the treatment of CRLM.^1^

Postoperative hepatic arterial infusion pump (HAIP) chemotherapy in combination with systemic chemotherapy has been demonstrated to reduce hepatic recurrences and improve overall survival (OS) in patients with resectable CRLM.^2,3^ HAIP chemotherapy is a liver-directed therapy that makes use of the arterial blood supply of CRLM and chemotherapeutic agents with a high first-pass effect to achieve maximal concentration of chemotherapeutic agents in the liver metastases with low systemic concentrations of chemotherapy.^4^ The potential benefit of HAIP chemotherapy is therefore to be expected in patients who are at risk of developing recurrence confined to the liver. Biomarkers are needed to better identify patients who are most likely to develop recurrence confined to the liver.

Histopathological growth patterns (HGPs) of CRLM are assessed at the tumor-liver interface of resected metastases on routine H&E slides via light microscopy. HGPs can be divided into desmoplastic HGP (dHGP), replacement HGP (rHGP), and pushing HGP (pHGP). dHGP is characterized by a separation between the tumor cells and hepatocytes by a rim of desmoplastic tissue, with no direct contact between the tumor and the liver parenchyma. rHGP shows direct contact between the tumor cells and the hepatocytes. The tumor cells replace the hepatocytes in the liver cell plates and the original architecture of the liver parenchyma is preserved. pHGP is characterized by direct contact between the tumor cells and the hepatocytes. Unlike in rHGP, there is no infiltrative growth. Instead, the surrounding liver cell plates show a compressed aspect.^5^ Patients can be divided into two clinically relevant groups based on the proportions of the HGPs found at the tumor-liver interface: patients with pure desmoplastic HGP (dHGP) and patients with any amount of non-dHGP, meaning replacement or pushing HGP.^6,7^ Patients with pure dHGP have an associated superior OS and progression-free survival (PFS) compared with patients with any amount of non-dHGP, regardless of the amount of non-dHGP, and independent of other independent clinicopathological prognostic factors such as BRAF and KRAS mutational status.^6,8,9^ In addition to the prognostic value, pure dHGP has been shown to primarily recur in the liver while non-dHGP has a higher rate of extrahepatic recurrence.^10^

Therefore, HGPs of CRLM may be suitable biomarkers for identifying patients that are predominantly at risk of developing hepatic recurrence after liver metastasectomy.^10^ This study investigated the predictive value of HGPs for adjuvant HAIP chemotherapy in patients with resectable CRLM.

## Methods

### Study Design and Patients

A retrospective cohort study was performed. Patients who underwent curative intent local therapy (i.e., resection and/or ablation) and perioperative systemic chemotherapy for CRLM at the Erasmus MC, Rotterdam and Memorial Sloan Kettering Cancer Center, New York between 1990 and 2019 were screened for inclusion. Patients were excluded if there was a history of extrahepatic disease (EHD) at, or prior to, the time of resection. Patients were excluded if HGPs were not assessed (tissue not available for analysis). Patients who underwent preoperative downstaging HAIP chemotherapy were excluded as well. Curative intent was defined as local treatment of all preoperatively identified lesions. Local treatment included surgical resection and local ablation.

HGPs were assessed retrospectively by at least two trained observers following the consensus guidelines.^6^ All observers were blinded to patient outcomes during HGP assessment. The HGP was scored as a percentage of the tumor-liver interface per H&E slide. The average HGP score of all slides was calculated by lesion and subsequently per patient. Patients were grouped into those with pure dHGP and those with any amount of non-dHGP.

Systemic chemotherapy consisted of a combination of fluorouracil (5-FU) infusion and bolus leucovorin (LV), oxaliplatin (OXA) and/or irinotecan-based chemotherapies. Patients who underwent HAIP chemotherapy underwent surgical pump implantation (combined with resection of CRLM) followed by up to 6 cycles of continuous HAIP floxuridine at 0.12 mg/kg/day, according to MSKCC protocol.^3^ All patients in the HAIP group received concomitant systemic chemotherapy.

Differences in baseline characteristics were compared using a chi-square exact test for percentages and Kruskal-Wallis test for medians of continuous data. Continuous variables are given as median with the interquartile range (IQR), unless indicated otherwise. OS was defined as the time in months from local treatment of liver metastases to death. PFS was defined as the time in months between local treatment for liver metastases and disease or death. Hepatic PFS (hPFS) was defined as the time in months from local treatment of liver metastases to hepatic progression or death (extrahepatic progression was not considered an event). Median follow-up for survivors was assessed using the reverse Kaplan–Meier method. OS, PFS, and hPFS were assessed via Kaplan–Meier analysis and compared using the log-rank test. Predictors of OS, PFS, and hPFS were assessed using uni- and multivariable Cox regression. Variables included in the initial Cox regression model were selected based on previous literature.^11^

A *p* value of < 0.05 was considered statistically significant. All analyses were performed in R version 4.0.2.^12^

## Results

### Patient Characteristics

**Fig. 1:**
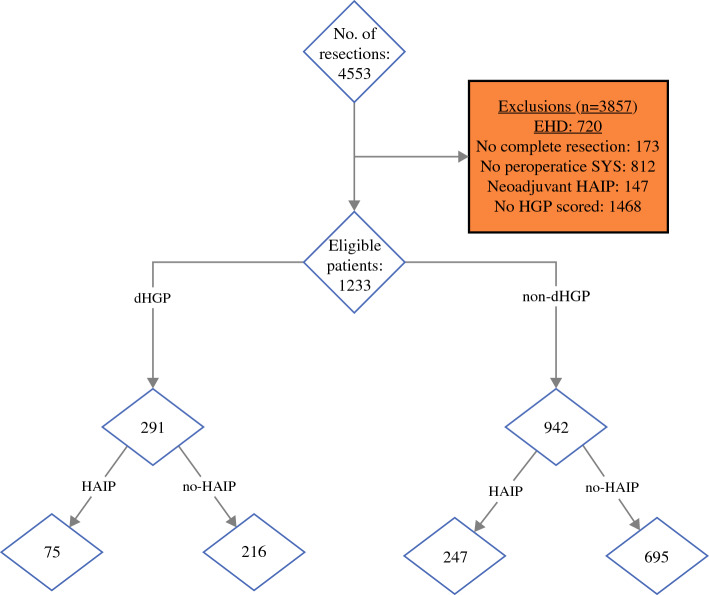
Inclusion flowchart

Between 1990 and 2019, 4553 patients underwent surgical treatment for CRLM. Patients were excluded due to a history of EHD (*n *= 720), incomplete resection of CRLM (*n *= 173), no perioperative systemic chemotherapy administered (*n *= 812), or HGP not analyzed (*n *= 1468). A total of 1233 patients were included, of whom 291 (24%) were dHGP patients and 942 (76%) were non-dHGP patients. In the dHGP group, 75 (26%) were treated with HAIP chemotherapy (HAIP group) versus 247 (26%) treated with HAIP in the non-dHGP group (Fig. 1).

Table 1 summarizes the baseline characteristics by HGP and HAIP treatment. Patients in the HAIP group were younger, had significantly worse tumor characteristics and a higher ASA score in both HGP groups. All patients undergoing HAIP chemotherapy were treated at MSKCC.

**Table 1:** Baseline characteristics in all patients (*n *= 1233)

	Missing data *n* (%)	dHGP			non-dHGP		
		HAIP (*n *= 75)	no HAIP (*n *= 216)	*p* value	HAIP (*n *= 247)	no HAIP (*n *= 695)	*p* value
Age, median [IQR]	–	53.0 [47.0, 61.0]	63.0 [52.0, 70.0]	**< 0.001**	54.0 [46.0, 65.0]	62.0 [53.0, 69.0]	**< 0.001**
Female gender, *n* (%)	–	50 (67)	140 (65)	0.772	159 (64)	411 (59)	0.148
ASA > II, *n* (%)	9 (1)	38 (51)	80 (37)	**0.044**	150 (61)	281 (42)	**< 0.001**
Location primary tumor, *n* (%)	55 (4.5)	–	–	0.099	–	–	0.812
Right-sided	–	23 (32)	55 (27)	–	58 (25)	166 (25)	–
Left-sided	–	37 (51)	90 (44)	–	115 (49)	315 (47)	–
Rectum	–	12 (17)	61 (30)	–	62 (26)	191 (28)	–
Disease-free Interval > 1 year, *n* (%)	–	7 (9)	32 (15)	0.230	87 (31)	54 (21)	0.008
Number of CRLM, median [IQR]	9 (1)	3.0 [2.0, 5.0]	1.0 [1.0, 2.0]	**< 0.001**	3.0 [1.0, 4.0]	2.0 [1.0, 3.0]	**< 0.001**
Diameter of CRLM, median [IQR]	33 (3)	2.2 [1.2, 3.2]	1.7 [1.1, 3.0]	0.433	2.9 [2.0, 4.9]	2.6 [1.7, 4.2]	**0.011**
Preoperative CEA, median [IQR]	148 (12)	7.0 [3.1, 19.0]	8.0 [3.1, 28.0]	0.699	12.1 [5.1, 47.0]	15.3 [4.6, 52.7]	0.567
Involvement of locoregional lymph nodes (N+)	12 (1)	10 (14)	22 (10)	0.446	214 (87)	566 (84)	0.260
Fong clinical risk score, *n* (%)	–	–	–	0.778	–	–	**0.009**
Low (0–2)	–	33 (44)	91 (42)	–	113 (46)	385 (55)	–
High (3–5)	–	33 (44)	91 (42)	–	134 (54)	310 (45)	–
KRAS mutation, *n* (%)	652 (53)	25 (41)	32 (42)	0.927	76 (37)	133 (45)	0.055
Preoperative systemic CTx	–	63 (84)	195 (90)	0.140	168 (68)	547 (79)	**0.001**
Involvement resection margin (R1), *n* (%)	12 (1)	10 (14)	22 (10)	0.446	36 (13)	35 (14)	0.152
Major hepatectomy, *n* (%)	80 (7)	30 (45)	71 (34)	0.108	117 (53)	272 (42)	**0.004**
Treatment center MSKCC, *n* (%)	0	75 (100)	111 (51)	**< 0.001**	247 (100)	417 (60)	**< 0.001**

Bold values are statistically significant (*p* < 0.05)

### Survival Outcomes of the Whole Cohort

The median follow-up period for survivors of the whole population was 71 months (IQR 38–106). During follow-up, 575 patients died, 810 patients had a recurrence, and 471 patients had a hepatic recurrence with or without the presence of extrahepatic recurrence.

The 5-year OS for the whole cohort was 55% (95% CI 52–58%); the 5-year PFS was 24% (95% CI 22–28%); and the 5-year hPFS for the whole cohort was 36% (95% CI 33–39%).

### Survival Outcomes in dHGP Group

**Fig. 2:**
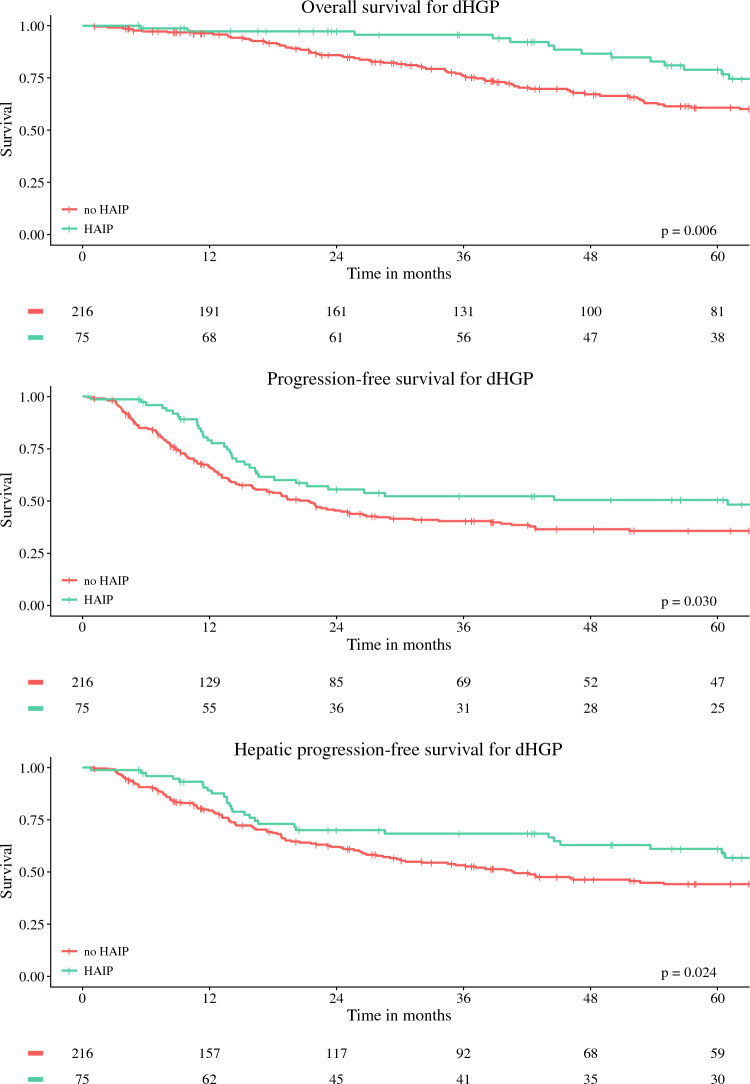
Kaplan–Meier figures for patients with dHGP

Survival curves for OS, PFS, and hPFS for dHGP patients according to treatment group are depicted in Fig. 2 and summarized in Table 2. For dHGP patients, the 5-year OS was 79% (95% CI 69–90%) for the HAIP group and 61% (95% CI 54–69%) for the no-HAIP group, *p *= 0.06. The 5-year PFS was 50% (95% CI 40–64%) for the HAIP group, versus 36% (95% CI 30–43%) for the no-HAIP group, *p *= 0.03; and the 5-year hPFS was 61% (95% CI 50–74%) for the HAIP group compared with 44% (95% CI 38–52%) for the no-HAIP group, *p* = 0.025.

**Table 2:** Five-year survival rates

Endpoint	dHGP		non-dHGP	
	HAIP	no HAIP	HAIP	no HAIP
5-year OS	79% (95% CI 69–90%)	61% (95% CI 54–69%)	60% (95% CI 53–67%)	49% (95% CI 44–66%)
5-year PFS	50% (95% CI 40–64%)	36% (95% CI 30–43%)	30% (95% CI 25–37%)	16% (95% CI 14–20%)
5-year hPFS	61% (95% CI 50–74%)	44% (95% CI 38–52%)	48% (95% CI 41–56%)	27% (95% CI 23–31%)

### Survival Outcomes in Non-dHGP Group

**Fig. 3:**
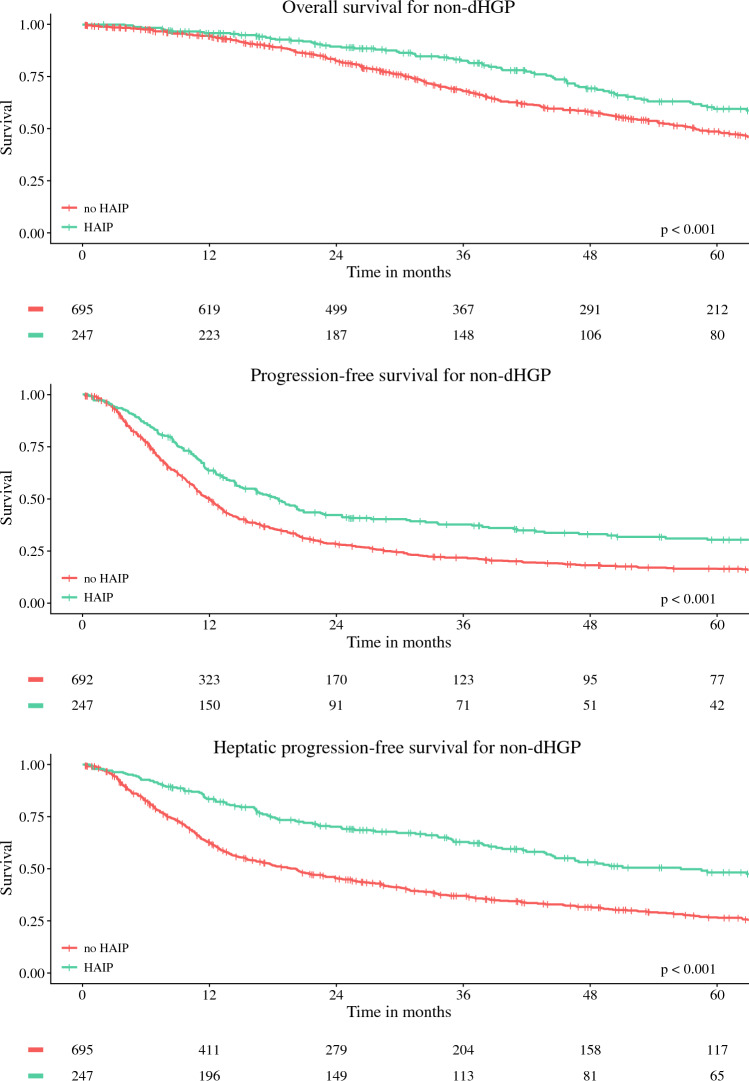
Kaplan–Meier figures for patients with non-dHGP

Survival curves for OS, PFS, and hPFS for dHGP patients according to treatment group are depicted in Fig. 3. In non-dHGP patients, the 5–year OS was 60% (95% CI 53–67%) for the HAIP and 49% (95% CI 44–66%) for the no-HAIP group, *p *< 0.001. Five-year PFS was 30% (95% CI 25–37%) for the HAIP group versus 16% (95% CI 14–20%) for the no-HAIP group, *p *< 0.001. hPFS was 48% (95% CI 41–56%) for the HAIP group compared with 27% (95% CI 23–31%) for the no-HAIP group, *p *< 0.001.

### Multivariable Regression Analysis

Complete case multivariate analysis was performed for 496 patients (40%). dHGP was associated with improved OS (HR 0.49 [0.32–0.73], *p *< 0.001), PFS (HR 0.72 [0.52–0.99], *p *= 0.049), and hPFS (HR 0.49 [0.32–0.73], *p *< 0.001). HAIP chemotherapy was associated with improved OS (HR 0.61 [0.45–0.82], *p *< 0.001), PFS (HR 0.47 [0.36–0.60], *p *< 0.001), and hPFS (HR 0.61 [0.45–0.82], *p *< 0.001) (Table 3). No statistically significant interaction between HAIP and HGP was found for OS (*p *= 0.40), PFS (*p *= 0.90), or hPFS (*p *= 0.46) when the interaction term was added to the model (Table 4).

**Table 3:** Cox regression analysis for OS, PFS, and hPFS without interaction terms

	Univariable	*p* value	Multivariable	*p* value
*Overall survival (n *= *496)*				
Age < 65 years	0.73 [0.62–0.86]	**< 0.001**	1.01 [0.73–1.39]	0.95
ASA > II	0.95 [0.80–1.13]	0.56	1.08 [0.79–1.46]	0.63
Location primary tumor				
Right-sided	Reference	–	Reference	–
Left-sided	0.84 [0.69–1.03]	0.10	0.83 [0.59–1.18]	0.31
Rectum	0.83 [0.66–1.04]	0.11	0.86 [0.57–1.29]	0.46
Preoperative systemic CTx	1.75 [1.40–2.19]	**< 0.001**	1.67 [1.13–2.47]	**< 0.01**
Number of CRLM	1.09 [1.06–1.12]	**< 0.001**	1.10 [1.04–1.16]	**< 0.01**
Diameter of largest CRLM (cm)	1.04 [1.02–1.07]	**< 0.01**	1.08 [1.03–1.14]	**< 0.001**
Preoperative CEA (before chemo)	1.00 [1.00–1.00]	0.76	1.00 [1.00–1.00]	0.63
Disease-free interval (months)	1.00 [0.99–1.00]	0.54	1.00 [0.98–1.01]	0.51
Nodal status primary tumor	1.45 [1.22–1.73]	**< 0.001**	2.01 [1.42–2.83]	**< 0.001**
R1 resection	1.68 [1.36–2.07]	**< 0.001**	1.53 [1.01–2.31]	**0.04**
KRAS mutated	1.71 [1.32–2.21]	**< 0.001**	1.63 [1.20–2.22]	**< 0.01**
dHGP (vs. non-dHGP)	0.67 [0.54–0.82]	**< 0.001**	0.49 [0.32–0.73]	**< 0.001**
HAIP chemotherapy	0.62 [0.50–0.76]	**< 0.001**	0.61 [0.45–0.82]	**< 0.001**
*Progression-free survival*				
Age < 65 years	0.97 [0.84–1.13]	0.73	1.29 [0.98–1.70]	0.07
ASA >II	0.92 [0.80–1.07]	0.29	1.32 [1.02–1.71]	**0.04**
Location primary tumor				
Right-sided	Reference	–	Reference	–
Left-sided	1.08 [0.90–1.28]	0.43	1.14 [0.84–1.54]	0.39
Rectum	1.07 [0.87–1.30]	0.52	1.25 [0.88–1.76]	0.21
Preoperative systemic CTx	1.83 [1.51–2.22]	**< 0.001**	1.34 [0.97–1.85]	0.08
Number of CRLM	1.13 [1.10–1.15]	**< 0.001**	1.16 [1.10–1.21]	**< 0.001**
Diameter of largest CRLM (cm)	1.01 [0.99–1.04]	0.31	1.05 [1.00–1.10]	**0.05**
Preoperative CEA (before chemo)	1.00 [1.00–1.00]	0.86	1.00 [1.00–1.00]	0.36
Disease-free interval (months)	0.99 [0.99–1.00]	**< 0.001**	0.99 [0.98–1.01]	0.28
Nodal status primary tumor	1.36 [1.17–1.58]	**< 0.001**	1.75 [1.33–2.31]	**< 0.001**
R1 resection	1.56 [1.29–1.89]	**< 0.001**	1.19 [0.82–1.73]	0.36
KRAS mutated	1.40 [1.14–1.73]	**< 0.01**	1.41 [1.09–1.82]	**< 0.01**
dHGP (vs. non-dHGP)	0.64 [0.53–0.76]	**< 0.001**	0.72 [0.52–0.99]	**0.05**
HAIP chemotherapy	0.53 [0.45–0.64]	**< 0.001**	0.47 [0.36–0.60]	**< 0.001**
*Hepatic progression*-*free survival*				
Age < 65 years	0.73 [0.62–0.86]	**< 0.001**	1.01 [0.73–1.39]	0.95
ASA >II	0.95 [0.80–1.13]	0.56	1.08 [0.79–1.46]	0.63
Location primary tumor				
Right-sided	Reference	–	Reference	–
Left-sided	0.84 [0.69–1.03]	0.10	0.83 [0.59–1.18]	0.31
Rectum	0.83 [0.66–1.04]	0.11	0.86 [0.57–1.29]	0.46
Preoperative systemic CTx	1.75 [1.40–2.19]	**< 0.001**	1.67 [1.13–2.47]	**< 0.01**
Number of CRLM	1.09 [1.06–1.12]	**< 0.001**	1.10 [1.04–1.16]	**< 0.01**
Diameter of largest CRLM (cm)	1.04 [1.02–1.07]	**< 0.01**	1.08 [1.03–1.14]	**< 0.001**
Preoperative CEA (before chemo)	1.00 [1.00–1.00]	0.76	1.00 [1.00–1.00]	0.63
Disease-free interval (months)	1.00 [0.99–1.00]	0.54	1.00 [0.98–1.01]	0.51
Nodal status primary tumor	1.45 [1.22–1.73]	**< 0.001**	2.01 [1.42–2.83]	**< 0.001**
R1 resection	1.68 [1.36–2.07]	**< 0.001**	1.53 [1.01–2.31]	**0.04**
KRAS mutated	1.71 [1.32–2.21]	**< 0.001**	1.63 [1.20–2.22]	**< 0.01**
dHGP (vs. non-dHGP)	0.67 [0.54–0.82]	**< 0.001**	0.49 [0.32–0.73]	**< 0.001**
HAIP chemotherapy	0.62 [0.50–0.76]	**< 0.001**	0.61 [0.45–0.82]	**< 0.001**

Bold values are statistically significant (*p* < 0.05)

**Table 4:** HGP-HAIP interaction added to multivariate analysis for OS, PFS, hPFS

	Univariable	*p* value	Multivariable	*p* value
*Overall survival (n = 496)*				
Age < 65 years	0.73 [0.62–0.86]	**< 0.001**	1.01 [0.73–1.39]	0.95
ASA > II	0.95 [0.80–1.13]	0.56	1.08 [0.80–1.47]	0.62
Location primary tumor				
Right-sided	Reference	–	Reference	–
Left-sided	0.84 [0.69–1.03]	0.10	0.83 [0.59–1.18]	0.30
Rectum	0.83 [0.66–1.04]	0.11	0.86 [0.57–1.29]	0.46
Preoperative systemic CTx	1.75 [1.40–2.19]	**< 0.001**	1.67 [1.13–2.47]	**< 0.01**
Number of CRLM	1.09 [1.06–1.12]	**< 0.001**	1.10 [1.04–1.16]	**< 0.01**
Diameter of largest CRLM (cm)	1.04 [1.02–1.07]	**< 0.01**	1.08 [1.03–1.14]	**< 0.001**
Preoperative CEA (before chemo)	1.00 [1.00–1.00]	0.76	1.00 [1.00–1.00]	0.63
Disease-free interval (months)	1.00 [0.99–1.00]	0.54	1.00 [0.98–1.01]	0.50
Nodal status primary tumor	1.45 [1.22–1.73]	**< 0.001**	2.01 [1.42–2.83]	**< 0.001**
R1 resection	1.68 [1.36–2.07]	**< 0.001**	1.52 [1.01–2.30]	**0.05**
KRAS mutated	1.71 [1.32–2.21]	**< 0.001**	1.64 [1.21–2.22]	**< 0.01**
dHGP (vs. non-dHGP)	0.71 [0.56–0.89]	**< 0.01**	0.47 [0.27–0.82]	**< 0.01**
HAIP chemotherapy	0.66 [0.52–0.83]	**< 0.001**	0.60 [0.44–0.83]	**< 0.01**
Interaction term: dHGP*HAIP	0.77 [0.44–1.32]	0.34	1.06 [0.47–2.36]	0.90
*Progression-free survival*				
Age < 65 years	1.01 [0.88–1.16]	0.84	1.32 [1.02–1.69]	**0.03**
ASA >II	0.95 [0.83–1.09]	0.46	1.11 [0.88–1.40]	0.38
Location primary tumor				
Right-sided	Reference	–	Reference	–
Left-sided	1.07 [0.90–1.27]	0.43	1.29 [0.97–1.70]	0.08
Rectum	1.12 [0.93–1.35]	0.21	1.40 [1.02–1.91]	**0.04**
Preoperative systemic CTx	1.65 [1.39–1.96]	**< 0.001**	1.50 [1.12–1.99]	**< 0.01**
Number of CRLM	1.11 [1.08–1.13]	**< 0.001**	1.15 [1.10–1.20]	**< 0.001**
Diameter of largest CRLM (cm)	1.04 [1.02–1.06]	**< 0.001**	1.08 [1.03–1.12]	**< 0.001**
Preoperative CEA (before chemo)	1.00 [1.00–1.00]	**0.04**	1.00 [1.00–1.00]	0.99
Disease-free interval (months)	0.99 [0.99–1.00]	**< 0.01**	0.99 [0.98–1.00]	0.22
Nodal status primary tumor	1.38 [1.20–1.59]	**< 0.001**	1.58 [1.24–2.02]	**< 0.001**
R1 resection	1.51 [1.26–1.82]	**< 0.001**	1.27 [0.91–1.77]	0.16
KRAS mutated	1.63 [1.35–1.97]	**< 0.001**	1.73 [1.37–2.18]	**< 0.001**
dHGP (vs. non-dHGP)	0.58 [0.48–0.70]	**< 0.001**	0.47 [0.32–0.70]	**< 0.001**
HAIP chemotherapy	0.65 [0.55–0.78]	**< 0.001**	0.50 [0.39–0.64]	**< 0.001**
Interaction term: dHGP*HAIP	1.01 [0.67–1.52]	0.95	1.29 [0.72–2.32]	0.40
*Hepatic progression-free survival*				
Age < 65 years	0.97 [0.84–1.13]	0.73	1.29 [0.98–1.71]	0.07
ASA >II	0.92 [0.80–1.07]	0.29	1.33 [1.03–1.73]	**0.03**
Location primary tumor				
Right-sided	Reference	–	Reference	–
Left-sided	1.08 [0.90–1.28]	0.43	1.14 [0.85–1.55]	0.38
Rectum	1.07 [0.87–1.30]	0.52	1.26 [0.89–1.78]	0.20
Preoperative systemic CTx	1.83 [1.51–2.22]	< 0.001	1.34 [0.97–1.85]	0.08
Number of CRLM	1.13 [1.10–1.15]	< 0.001	1.15 [1.10–1.21]	**< 0.001**
Diameter of largest CRLM (cm)	1.01 [0.99–1.04]	0.31	1.05 [1.00–1.10]	**0.05**
Preoperative CEA (before chemo)	1.00 [1.00–1.00]	0.86	1.00 [1.00–1.00]	0.36
Disease-free interval (months)	0.99 [0.99–1.00]	< 0.001	0.99 [0.98–1.00]	0.26
Nodal status primary tumor	1.36 [1.17–1.58]	< 0.001	1.76 [1.33–2.32]	**< 0.001**
R1 resection	1.56 [1.29–1.89]	< 0.001	1.17 [0.80–1.71]	0.41
KRAS mutated	1.40 [1.14–1.73]	< 0.01	1.42 [1.10–1.83]	**< 0.01**
dHGP (vs. non-dHGP)	1.67 [1.37–2.04]	< 0.001	1.53 [1.01–2.32]	**0.05**
HAIP chemotherapy	0.63 [0.42–0.94]	0.02	0.57 [0.32–1.01]	**0.05**
Interaction term: dHGP*HAIP	0.80 [0.51–1.24]	0.31	0.79 [0.42–1.49]	**0.46**

Bold values are statistically significant (*p* < 0.05)

## Discussion

No interaction between HGP and adjuvant HAIP chemotherapy was found in patients who underwent resection of CRLM. HAIP was associated with an improved OS, PFS, and hPFS, regardless of HGP.

Previous studies have shown improved OS associated with HAIP chemotherapy in patients with CRLM and a reduced rate of hepatic recurrences.^3,13–15^ In a retrospective series of 2368 patients with resectable CRLM, the median survival was 67 months with, versus 44 months without, HAIP chemotherapy (HR: 0.67 [95% CI 0.59–0.76], *p* < 0.001).^14^ Moreover, long-term results of a randomized controlled trial showed a HAIP chemotherapy-associated improvement in 2-year OS and PFS (31.3 vs. 17.2 months, *P* = 0.02) in patients with resectable CRLM.^13^ It is likely that not all patients with resected CRLM benefit equally from HAIP chemotherapy. For example, the retrospective study found that patients with extrahepatic disease, whether or not resected, did not appear to benefit from HAIP chemotherapy.^14^ More biomarkers are needed to select patients who are most likely to benefit from HAIP chemotherapy.

Previous studies have shown that CRLM with a non-dHGP were associated with an increased risk of multiorgan recurrence compared with patients with dHGP.^10^ Buisman et al. found that postoperative systemic chemotherapy was associated with a an improved PFS in the non-dHGP group, but not in the dHGP group, albeit only in patients who have not undergone preoperative systemic chemotherapy.^11^ A potential explanation could be the more aggressive tumor biology associated with non-dHGP, and subsequent higher recurrence risk outside the liver.^5,16–18^ Previous studies also found that patients with dHGP were more likely to recur in the liver only.^5^ We therefore hypothesized that HAIP-associated survival benefit would be more pronounced in dHGP patients. However, we could not demonstrate that HGP was predictive of the effectiveness of adjuvant HAIP for resectable CRLM. What is more, HAIP patients in the dHGP group showed a similar hPFS to the HAIP group in the non-dHGP group, while OS and PFS were lower in the latter group. This further supports our findings that HAIP effectively prevents hepatic recurrence regardless of HGP, while non-dHGP patients are more likely to develop (and succumb to) extrahepatic progression.

The lack of interaction between HGP and HAIP chemotherapy in hPFS suggests that the differences in tumor biology do not predict the effectiveness of HAIP chemotherapy. However, the effect of tumor HGPs on HAIP efficacy are unknown and should be considered in future research.

This study has certain limitations. Firstly, the retrospective nature poses a high risk of selection bias between HAIP and no-HAIP treatment. There are some differences in baseline characteristics between HAIP and no-HAIP patients in both HGP groups; HAIP patients were younger, but had overall more unfavorable tumor characteristics in both HGP groups. All HAIP patients were treated at MSKCC. HAIP remained an independent favorable prognostic factor for OS, PFS, and hPFS. Nevertheless, ideally the effect of HGP on the efficacy of HAIP chemotherapy should be investigated in a randomized controlled trial powered for OS. Currently, a large Dutch randomized controlled trial (the PUMP trial) is investigating the effect of resection alone versus resection and postoperative HAIP chemotherapy in patients with CRLM and a low MSKCC clinical risk score.^9,19,20^ In addition, HGPs were available for analysis in a small number of patients, leading to further selection bias and a sample size too small to detect a statistically significant difference in OS, particularly in the dHGP group. Another limitation is that a significant proportion of patients (79%) underwent neoadjuvant systemic chemotherapy, which is strongly suspected to induce histopathological changes in the CRLM that lead to an increase in dHGP, and thus lead to a reduced prognostic value of HGP.^21^

In conclusion, we confirmed that dHGP and HAIP chemotherapy were associated with improved survival, but could not demonstrate that HGP is a useful biomarker to select patients for adjuvant HAIP chemotherapy after resection of CRLM.

## References

[1] Tomlinson JS, Jarnagin WR, DeMatteo RP, Fong Y, Kornprat P, Gonen M (2007). Actual 10-year survival after resection of colorectal liver metastases defines cure. J Clin Oncol..

[2] Groot Koerkamp B, Hunink MG, Stijnen T, Hammitt JK, Kuntz KM, Weinstein MC (2007). Limitations of acceptability curves for presenting uncertainty in cost-effectiveness analysis. Med Decis Making..

[3] Kemeny N, Huang Y, Cohen AM, Shi W, Conti JA, Brennan MF (1999). Hepatic arterial infusion of chemotherapy after resection of hepatic metastases from colorectal cancer. New Engl J Med..

[4] Ensminger WD, Rosowsky A, Raso V, Levin DC, Glode M, Come S (1978). A clinical-pharmacological evaluation of hepatic arterial infusions of 5-fluoro-2'-deoxyuridine and 5-fluorouracil. Cancer Res..

[5] Vermeulen PB, Colpaert C, Salgado R, Royers R, Hellemans H, Van Den Heuvel E (2001). Liver metastases from colorectal adenocarcinomas grow in three patterns with different angiogenesis and desmoplasia. J Pathol..

[6] Latacz E, Höppener D, Bohlok A, Leduc S, Tabariès S, Moro CF (2022). Liver metastases from colorectal adenocarcinomas grow in three patterns with different angiogenesis and desmoplasia. Br J Cancer..

[7] van Dam PJ, van der Stok EP, Teuwen LA, Van den Eynden GG, Illemann M, Frentzas S (2017). International consensus guidelines for scoring the histopathological growth patterns of liver metastasis. Br J Cancer..

[8] Galjart B, Nierop PMH, van der Stok EP, van den Braak R, Höppener DJ, Daelemans S (2019). Angiogenic desmoplastic histopathological growth pattern as a prognostic marker of good outcome in patients with colorectal liver metastases. Angiogenesis..

[9] Höppener DJ, Galjart B, Nierop PMH, Buisman FE, van der Stok EP, Coebergh van den Braak RRJ (2021). Histopathological growth patterns and survival after resection of colorectal liver metastasis: an external validation study. JNCI Cancer Spectr..

[10] Nierop PMH, Galjart B, Hoppener DJ, van der Stok EP, Coebergh van den Braak RRJ, Vermeulen PB (2019). Salvage treatment for recurrences after first resection of colorectal liver metastases: the impact of histopathological growth patterns. Clin Exp Metastasis..

[11] Buisman FE, van der Stok EP, Galjart B, Vermeulen PB, Balachandran VP, Coebergh van den Braak RRJ (2020). Histopathological growth patterns as biomarker for adjuvant systemic chemotherapy in patients with resected colorectal liver metastases. Clin Exp Metast..

[12] Team RC. R: A language and environment for statistical computing. 4.0.2 ed 2020.

[13] Kemeny NE, Gonen M (2005). Hepatic arterial infusion after liver resection. N Engl J Med.

[14] Koerkamp BG, Sadot E, Kemeny NE, Gönen M, Leal JN, Allen PJ (2017). Perioperative hepatic arterial infusion pump chemotherapy is associated with longer survival after resection of colorectal liver metastases: a propensity score analysis. J Clin Oncol..

[15] Koerkamp BG, Sadot E, Kemeny NE, Gönen M, Leal JN, Allen PJ (2022). Adjuvant intra-arterial chemotherapy for patients with resected colorectal liver metastases: a systematic review and meta-analysis. HPB..

[16] Frentzas S, Simoneau E, Bridgeman VL, Vermeulen PB, Foo S, Kostaras E (2016). Vessel co-option mediates resistance to anti-angiogenic therapy in liver metastases. Nat Med..

[17] Stessels F, Van den Eynden G, Van der Auwera I, Salgado R, Van den Heuvel E, Harris AL (2004). Breast adenocarcinoma liver metastases, in contrast to colorectal cancer liver metastases, display a non-angiogenic growth pattern that preserves the stroma and lacks hypoxia. Br J Cancer..

[18] van Dam PJ, Daelemans S, Ross E, Waumans Y, Van Laere S, Latacz E (2018). Histopathological growth patterns as a candidate biomarker for immunomodulatory therapy. Semin Cancer Biol..

[19] Buisman FE, Homs MYV, Grünhagen DJ, Filipe WF, Bennink RJ, Besselink MGH (2019). Adjuvant hepatic arterial infusion pump chemotherapy and resection versus resection alone in patients with low-risk resectable colorectal liver metastases — the multicenter randomized controlled PUMP trial. BMC Cancer..

[20] Fong Y, Fortner J, Sun RL, Brennan MF, Blumgart LH (1999). Clinical score for predicting recurrence after hepatic resection for metastatic colorectal cancer: analysis of 1001 consecutive cases. Ann Surg..

[21] Nierop PM, Höppener DJ, Buisman FE, van der Stok EP, Galjart B, Balachandran VP (2022). Preoperative systemic chemotherapy alters the histopathological growth patterns of colorectal liver metastases. J Pathol Clin Res..

